# Train Your Brain? Can We Really Selectively Train Specific EEG Frequencies With Neurofeedback Training

**DOI:** 10.3389/fnhum.2020.00022

**Published:** 2020-03-10

**Authors:** Emilie Dessy, Olivier Mairesse, Martine van Puyvelde, Aisha Cortoos, Xavier Neyt, Nathalie Pattyn

**Affiliations:** ^1^VIPER Research Unit, Royal Military Academy, Brussels, Belgium; ^2^Experimental and Applied Psychology, Vrije Universiteit Brussel, Brussels, Belgium; ^3^Sleep Laboratory & Unit for Chronobiology U78, Brugmann University Hospital, Free University of Brussels-Université Libre de Bruxelles, Brussels, Belgium; ^4^Clinical and Lifespan Psychology, Faculty of Psychology and Educational Sciences, Vrije Universiteit Brussel, Brussels, Belgium; ^5^Department of Human Physiology, Vrije Universiteit Brussel, Brussels, Belgium

**Keywords:** neurofeedback training, cognitive enhancement, performance, EEG frequencies, EEG changes, training specificity

## Abstract

Neurofeedback (NFB) is an operant conditioning procedure whereby an individual learns to self-regulate the electrical activity of his/her brain. Initially developed as a treatment intervention for pathologies with underlying EEG dysfunctions, NFB is also used as a training tool to enhance specific cognitive states required in high-performance situations. The original idea behind the NFB training effect is that the changes should only be circumscribed to the trained EEG frequencies. The EEG frequencies which are not used as feedback frequencies should be independent and not affected by the neurofeedback training. Despite the success of sensorimotor rhythm NFB training in cognitive performance enhancement, it remains unclear whether all participants can intentionally modify the power densities of specifically selected electroencephalographic (EEG) frequencies. In the present study, participants were randomly assigned to either a control heart rate variability (HRV) biofeedback (HRV) training group or a combination of HRV biofeedback and neurofeedback (HRV/NFB) training group. This randomized mixed design experiment consisted of two introductory theoretical lessons and a training period of 6 weeks. We investigated the evolution of the different EEG frequency bands of our two experimental groups across and within session. All the participants exhibited EEG changes across and within session. However, within the HRV/NFB training group, untrained EEG frequencies have been significantly modified, unlike some of the trained frequencies. Moreover, EEG activity was modified in both the HRV group and the HRV/NFB groups. Hence, the EEG changes were not only circumscribed to the trained frequency bands or to the training modality.

## Introduction

Electroencephalographic neurofeedback (NFB), a modality of biofeedback based on the voluntary training of ongoing brain activity, has been extensively studied over the last decades. An important body of evidence suggests that NFB might be a promising technique within clinical and non-clinical populations. Within clinical populations, NFB assumes that pathological brain activity patterns associated with a specific clinical disorder, can be voluntarily modulated by the patient through operant learning strategies to “normalize” the brain activity, which then would lead to an improved cognitive and/or behavioral functioning [for a review see Niv (2013), Dessy et al. (2018)]. Within non-clinical populations, NFB is a nootropic using operant conditioning to train specific brainwave patterns associated with optimal cognitive functioning [for a review see Hammond et al. (2011)]. However, some researchers recently expressed scepticism about the published results supporting the NFB efficacy and emphasized the need for more rigorous studies (Thibault and Raz, 2016a, b, 2017, 2018; Thibault et al., 2017, 2018; Ros et al., 2019). To date, the main shortcomings of the existing studies include the scarcity of randomized and well-controlled trials (i.e., non-blinded participants and raters), and the absence of objective neuropsychological or electrophysiological data (Schönenberg et al., 2017; Ros et al., 2019). Indeed, a randomized, double-blind, sham-controlled milestone study from Schabus et al. (2017) showed that NFB may work for very different reasons than what the mainstream interpretations describe. Their results showed that the effect of NFB was only related to placebo effects and only showed in the participants’ subjective data (Schabus et al., 2017). Moreover, there is a lack of standard protocols with a well-defined number of sessions within the neurofeedback literature (Thibault and Raz, 2016a, b, 2017; Thibault et al., 2017; Ros et al., 2019).

To optimize performance, many of the applied protocols focus on sensorimotor rhythm (SMR) training, where the frequency band between 12 and 15 Hz at EEG sites above the sensorimotor cortex is trained to be enhanced (Dessy et al., 2018). One of the first significant controlled study in this context was published by Ros et al. (2009) who investigated the effect of NFB training on fine-psychomotor skills. The NFB training protocol consisted of eight training sessions in which participants had to increase the sensorimotor rhythm (SMR; 12–15 Hz) while simultaneously inhibiting theta (4–8 Hz) and high beta (21–30 Hz), all frequencies being measured across the sensorimotor strip (C3, Cz et C4). Results showed that SMR power correlated with inhibition of motor output and sensory input (Ros et al., 2009). The authors claimed that their NFB training protocol aimed at teaching people to put themselves in a calm, open and balanced psychological state (Ros et al., 2009). To preserve performance under stress, Sanders (1983) described the need for a well-developed “functional reserve,”, which previous authors (Pattyn et al., 2010, 2014) correlated with the individual’s normal range of variability of the autonomous nervous system (ANS) (Baevsky et al., 2005). This type of autonomic flexibility has been described in the neurovisceral integration model (e.g., Friedman, 2007), which identified a flexible neural network associated with self-regulation and adaptability that might help the organism to respond effectively to demands from the environment. In their studies on psychophysiological responses to operational stress, Pattyn et al. (2008, 2010, 2014) showed correlations between both disrupted control of attention and decreased cognitive performance on the one hand, and cardiac reactivity in response to a challenging stress situation on the other hand, where the highest reactivity correlated with the best performance (Pattyn et al., 2008, 2010, 2014). Thayer et al. (2009) suggested an association between higher levels of resting heart rate variability (HRV) and superior performance on tasks that demand executive functions (Thayer and Lane, 2009; Thayer et al., 2009). Thus, according to these authors, HRV reflects the cardiovascular system’s ability to adapt to external environmental challenges under the influence of the ANS (Thayer and Lane, 2009; Thayer et al., 2009). It is this cardiovascular flexibility which is targeted by the HRV biofeedback training (Lehrer et al., 2000; Lehrer and Gevirtz, 2014) through the voluntary control of respiration.

In a previous paper, we investigated in a quasi-randomized controlled design the effect of HRV biofeedback and neurofeedback training on cognitive and fine-psychomotor performances in a real-life stress period (Dessy et al., submitted). Our results showed no specific effect of the training on performance. However, our results showed significant cardio-respiratory modifications between the baseline and the stress period within both our experimental and control groups (Dessy et al., submitted). Whereas voluntary modifications of autonomic activation through the control of respiration require no additional demonstration (Wilmore and Costill, 2004; Guyenet, 2014), to date, it remains unclear whether participants can intentionally modify power densities of specifically selected electroencephalographic frequencies.

Indeed, self-regulation of peripherally measured physiological responses, such as electrocardiography, galvanic skin response, skin temperature or pupillary diameter are part of numerous clinical and non-clinical protocols (Yucha and Montgomery, 2008). However, the main question pertaining to EEG NFB training remains whether we are capable of perceiving and thus altering our EEG activity. To advance our understanding of the NFB mechanisms on brain function and behavior, a consortium of researchers established a standardized checklist outlining best practices in the experimental design and reporting of neurofeedback studies (Ros et al., 2019). It appears that learning indices, i.e., changes in the power densities of the targeted EEG frequency bands within and across training sessions, are often lacking in NFB studies (Thibault and Raz, 2016a, b, 2017; Thibault et al., 2017). This lack might explain discrepancies in reported efficacy of NFB training in the literature (Thibault and Raz, 2016a, b, 2017; Thibault et al., 2017; Ros et al., 2019) as the NFB-specific mechanisms cannot be disentangled from the other unspecific mechanisms driving the potential benefits of the NFB training. Additionally, information on frequency-specific modifications (i.e., circumscribed to the trained bands) and training (i.e., the association between trained bands and behavioral effects is well-defined) is scarcely present in most of the published investigation (Thibault and Raz, 2016a, b, 2017; Thibault et al., 2017). In the present study, we attempt to shed some light on this debate by specifically investigating the learning indices in a previously validated NFB training protocol (Ros et al., 2009). Our research question was whether NFB training can selectively modify specific EEG frequencies. Additionally, by comparing two experimental groups, i.e., a BFB training group and a combined BFB/NFB training group, we would like to assess training specificity and the potential unspecific mechanisms playing a role in our study.

## Materials and Methods

### Participants

Participants were all engineering students from the University of Stuttgart enrolled in a Soyuz spacecraft simulator training course at the Institute of Space Systems (IRS) (*N* = 26). The participants were randomly assigned to an HRV biofeedback training group (HRV; *N* = 11) or a combined HRV BFB/NFB training group (HRV/NFB; *N* = 15). Due to the small number of females and left-handed individuals, only right-handed male participants (age range 20 to 26) were included in this study to avoid additional confounds. All participants were German native speakers with a good passive and active level of English. All students participated voluntarily and signed an informed consent form. They all had normal or corrected-to-normal vision, did not report any medical condition in their medical questionnaires and were unmedicated. The study was approved by the Medical Ethics Committee of the University Hospital (UZ-Brussel) and the Vrije Universiteit Brussel (VUB) (B.U.N.: 143201319026, 2013/347).

### Design

**FIGURE 1:**
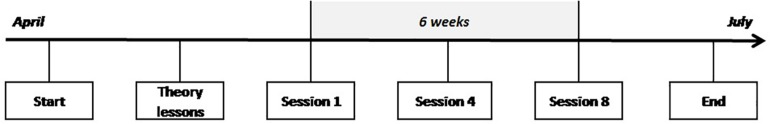
Time course of the study. The study took place for all participants from April to June. After following theory classes, participants followed eight training sessions over a 6 weeks period. Three data collection points of the training were considered for each participant: session 1, session 4, and session 8.

The participants were randomly assigned to either a BFB group (BFB) or a combined BFB/NFB training group (BFB/NFB). This study consisted of two introductory theoretical lessons and a training period of 6 weeks. To investigate the effect of the NFB training on electroencephalographic (EEG) data, three data collection points of the training were taken into account for each participant of the two experimental groups: training session 1 (start of the training), training session 4 (mid-training) and training session 8 (last training session) (see Figure 1).

### Procedure

**FIGURE 2:**
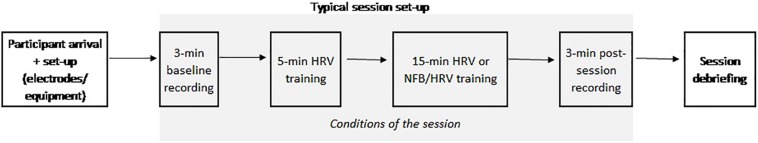
Typical sequence of a training session. After the set-up of the participant, one 3 min eyes open baseline was recorded at rest for participants of both groups. At the start of the training, they all followed a 5 min heart rate variability (HRV) biofeedback (BFB) training. Then, participants from the HRV group underwent a 15 min HRV training while the HRV/NFB group received a 15 min combined neurofeedback (NFB) and HRV BFB training. At the end of the training, one 3 min eyes open post-training recoding occurred. Each training session finished with a debriefing about the session between the participant and the experimenter.

All the participants followed two theory lessons before starting the 6-week training period. The first lesson introduced the human nervous system at a macro- and micro-level. The second one presented an introduction to BFB and NFB. Then, participants were randomly distributed into two groups (i.e., HRV group and HRV/NFB group). The HRV-group received eight HRV BFB training-sessions and the HRV/NFB-group eight combined HRV/NFB training sessions. The training sessions were scheduled for each participant weekly at a similar time slot (e.g., every week at 9 a.m. on Monday and Wednesday) to avoid circadian confounds over the different sessions. Training began and finished with a 3 min period where EEG-band powers were recorded at rest with eyes open, in the absence of feedback. Then, both the HRV and the HRV/NFB groups had to sustain a slow-paced rhythm of breathing at approximately six cycles per minute (cpm). Moreover, the HRV/NFB group was instructed to increase sensorimotor (SMR) power (12–15 Hz) and inhibit both theta power (4–8 Hz) and high beta power (21–30 Hz) at Cz. Participants were instructed to ask questions, help or breaks at their convenience. When needed, they were guided by the instructor to reflect on the encountered problems and how to overtake them. At the end of each training session, the participants gave feedback about the training (see Figure 2).

### Electrophysiology

The respiratory, electrocardiographic (ECG) and electroencephalographic (EEG) signals were recorded using a 10-channel amplifier (NeXus-10 MKII, Mind Media BV, Herten, Netherlans). EEG signals were sampled at 256 Hz with Ag/AgC electrodes at the central motor cortex (electrode site Cz) referenced and grounded on either earlobe. The signal was filtered with a 0.5 Hz high-pass and a 50 Hz low-pass filter and root mean squared in 1/8 s epochs in three frequency bands: SMR (12–15 Hz), high beta (21–30 Hz), and theta (4–8 Hz). All data were visually inspected offline and manually corrected for artifacts (e.g., muscle activity, eye movements) detections. Indeed, it has been required to filter out the significant artifacts from the EEG signals (less than 20% of the signal) such as facial EMG and ocular EMG activity (e.g., eye blinks and movements). After artifacts removal, EEG signals were analyzed through the proprietary software BioTrace + (Mind Media BV, Netherland). The EEG data were processed with fast Fourier transformation (FFT) to determine the power density of each frequency band in microvolt squared per hertz: theta (4–8 Hz), alpha (8–12 Hz), SMR (12–15 Hz), beta (15–21 Hz), and high beta (21–30 Hz).

### Biofeedback and Neurofeedback Training Modalities

**FIGURE 3:**
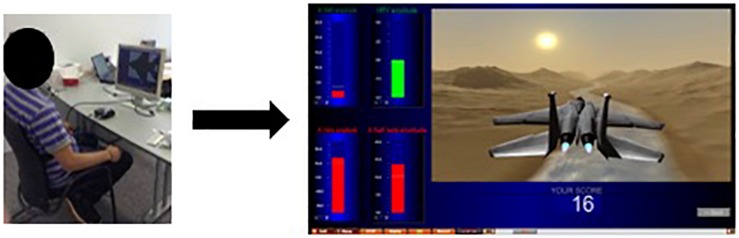
Typical set-up of a participant for the combined neurofeedback (NFB) and HRV BFB training session.

The BFB and NFB paradigms were generated through the proprietary software BioTrace + (Mind Media BV, Netherland). As stated in the procedure, both the HRV and the HRV/NFB groups had to sustain a slow-paced rhythm of breathing [six cycles per minute (cpm)] to increase their RSA amplitude which was computed in real-time using the root mean square of the standard deviation (Mind Media BV, Netherland). Moreover, the BFB/NFB group were instructed to increase sensorimotor (SMR) power (12–15 Hz) and inhibit both isolated theta power (4–8 Hz) and high beta power (21–30 Hz) at Cz. Everyone had access to a training screen displayed on a monitor placed in front of the participant (see Figure 3). This training screen displayed for the HRV/NFB group four bars graphs representing the power of the three different EEG frequency bands powers and of the RSA amplitude, visual feedback and a score counter. For the HRV group, it displayed only the RSA amplitude bar graph and the visual feedback. Whenever the band power reached an individually predefined threshold, the color of the bar graphs turned green and red (see Figure 3). The target values (i.e., feedback thresholds) were based on measurements during the first 2 min of each training session. Average thresholds were automatically calculated for each participant and session, hence tailored to the individual and the situation. The feedback modality was set up to reward the participants if HRV and SMR were maintained above the customized threshold and theta and high beta below the customized threshold. These thresholds were determined as 60% of the value of the respective frequency band during the two first minutes of recording. Subjects continuously received visual feedback (i.e., a video loop of a jet plane, a spaceship) which was changed at the request of the participant to counteract boredom, motivational decline and maximize participant engagement to perform. This visual feedback loop was displayed as a reward while the counter score increased when participants reached the stated training objective (i.e., parameter below or above threshold) (see Figure 3).

### Statistical Analysis

All statistical analyses were performed using version 25 of the Statistical Package for Social Sciences (SPSS 25, Chicago, IL, United States). The dependent variables were the power densities of the EEG frequency bands (expressed in microvolts squared per hertz, i.e., μV^2^/Hz) which were targeted by the training, either to increase (i.e., SMR) or to decrease (i.e., theta and high beta) their respective power or the untrained frequencies (i.e., alpha and beta). The independent variables were the sessions (i.e., sessions 1, 4 or 8), the session conditions (i.e., pre-training, HRV training, HRV/NFB training, post-training) and the groups (HRV or HRV/NFB). To respond to the research questions investigating the effect of the HRV and HRV/NFB training on the electroencephalographic data (effect of conditions) as well as the training efficacy (effect of sessions), the results of the measures obtained during the training sessions 1, 4, and 8 were tested using a 3 [sessions (session 1, session 4, session 8)] × 3 [conditions (pre-training, HRV training, HRV/NFB training or post-training)] × 2 [groups (HRV, HRV/NFB)] mixed ANOVA with sessions and conditions as within-subjects factors and groups as between subjects factor. Moreover, the results of the measures obtained for the HRV BFB training group during the training sessions 1, 4, and 8 were tested using a 3 [sessions (session 1, session 4, session 8)] × 3 [conditions (pre-training session, HRV training session, post-training session)] ANOVA with sessions and conditions as within-subjects factor. The results of the measures obtained for the HRV/BFB training group during the training sessions 1, 4 and 8 were tested using a 3 [sessions (session 1, session 4, session 8)] × 4 [conditions (pre-training session, HRV training session, HRV/NFB training, post-training session)] ANOVA with sessions and conditions as within-subjects factor. Greenhouse-Geisser Epsilon corrections were used when sphericity was violated to control for type 1 error associated with this type of violation. For all statistical tests, significant *p*-value level was set at *p* < 0.05.

## Results

To investigate if we can selectively train specific EEG frequencies with neurofeedback training, we analyzed first the results obtained for the HRV/NFB group, both across the different sessions (i.e., throughout the 6 weeks training) and conditions within a session (i.e., over 45 min) for the trained (i.e., theta, SMR and high beta) and untrained EEG frequency bands (i.e., alpha and beta). Our graphs emphasized the effect of these different conditions on the trained and untrained EEG frequencies across the different training sessions. Then, we examined the EEG results obtained for the HRV group across the different sessions and conditions within a session. The graphs depicted the effect of the HRV BFB training on the EEG frequencies across sessions. At last, we compared both groups across and within sessions. Only the significant results demonstrating a group effect are reported.

### Electroencephalographic Results

#### HRV/NFB Group

**FIGURE 4:**
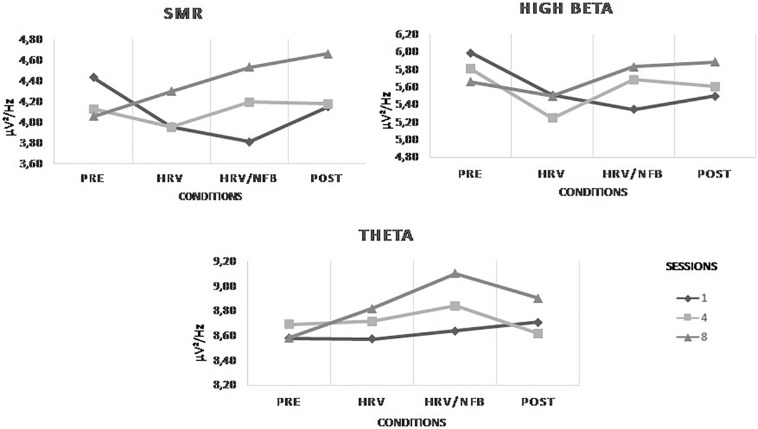
Mean power densities of the trained EEG frequency bands (i.e., SMR, high beta and theta) across conditions (i.e., pre, HRV, HRV/NFB, and post) within a session and across sessions (i.e., session 1, session 4, and session 8) for the combined HRV/NFB group. The only significant effect for the SMR was the interaction between session and condition.

Figure 4 depicts the results. Statistically, we observed no main effect of conditions or sessions on the trained EEG frequency bands at Cz. There is no significant effect of the HRV/NFB training within one session, nor across eight training sessions. Yet, there is a significant interaction between sessions and conditions for SMR power density [*F*(2.514,35.197) = 6.593, *p* = 0.002]. *Post hoc* analysis showed that SMR power density from session 8 compared to session 1 were significantly higher in the HRV/NFB condition than in the HRV condition and even higher in the post condition than in the HRV/NFB condition (see Figure 4).

**FIGURE 5:**
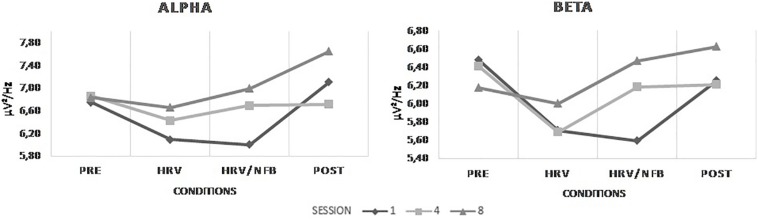
Mean power densities of the untrained EEG frequency bands (i.e., alpha and beta) across conditions (i.e., pre, HRV, HRV/NFB and post) within a session and across sessions (i.e., session 1, session 4, and session 8) for the combined HRV/NFB group. Alpha power density was significantly higher in session 8 than in session 1 as well as across condition independently of the session. Beta power density was significantly increasing across the different condition as well as across the training sessions.

With regards to the effect of the combined HRV/NFB training on the untrained EEG frequency bands at Cz, Figure 5 depicts the results. Statistically, there is a main effect of sessions on alpha power density [*F*(2,28) = 4.596, *p* = 0.019]. Alpha power density was significantly higher in session 8 than in session 1 (see Figure 5). Moreover, there is a main effect of conditions on alpha [*F*(3,28) = 5.034, *p* = 0.005] and beta [*F*(1.835,25.696) = 5.758, *p* = 0.01] power densities. Alpha and beta power densities were significantly increasing across the different conditions of the training session (see Figure 5). There is also significant interaction between sessions and conditions for beta power density [*F*(3.088,43.232) = 4.364, *p* = 0.009]. *Post hoc* analysis showed that, as for the results observed for the SMR frequency band, beta power density from session 8 compared to session 1 were significantly higher in the HRV/NFB condition than in the HRV condition and even higher in the post condition than in the HRV/NFB condition (see Figure 5).

#### HRV Group

**FIGURE 6:**
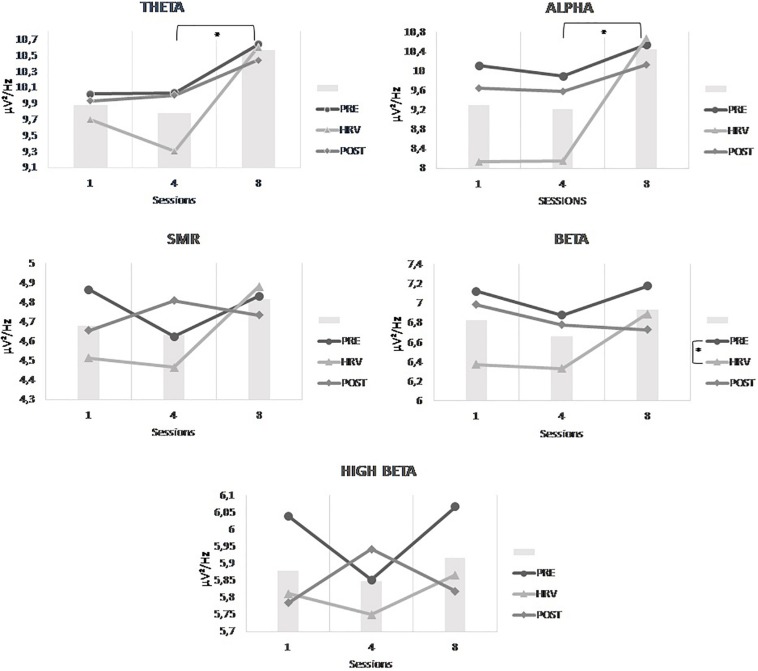
Mean power densities of the untrained EEG frequency bands (i.e., theta, alpha, SMR, beta, and high beta) across conditions (i.e., pre, HRV and post) within a session and across sessions (i.e., session 1, session 4, and session 8) for the HRV group. The bar graphs represent the mean power densities of the untrained EEG frequency bands across sessions, independently of the conditions. Significant main effects are highlighted on the respective graphs.

With regards to the effect within one session of the HRV training on the EEG activity at Cz, results are depicted in Figure 6. Statistically, there is a main effect of conditions on beta [*F*(2,20) = 4.430, *p* = 0.026] which is indicated in the *post hoc* analysis by a significantly higher beta power density in the pre-condition than in the HRV training condition (see Figure 6). Moreover, the effect of the HRV training is also present across the sessions. Indeed, there is a main effect of sessions on theta [*F*(2,20) = 7.373, *p* = 0.004] and a trend is observed for alpha [*F*(1.280, 17.804) = 4.224, *p* = 0.053]. *Post hoc* analysis showed that theta and alpha power densities were both significantly higher in session 8 than in session 4 (see Figure 6). However, there was no significant interaction between conditions and sessions.

#### Comparison of Both Groups

**FIGURE 7:**
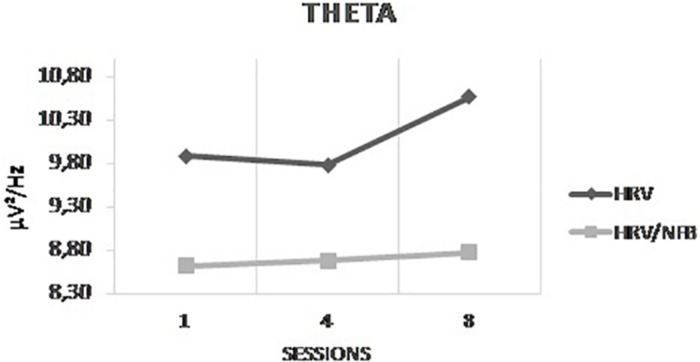
Mean power density of theta frequency band across sessions (i.e., session 1, session 4, and session 8) for both the HRV and HRV/NFB groups which is the only significant difference between both groups.

The results of the 3 × 3 × 2 Mixed ANOVA with sessions and conditions as within-subjects factors and groups as between-subjects factor yielded only a significant interaction between sessions and groups for theta [*F*(2,48) = 7.373, *p* = 0.035]. *Post hoc* analysis showed that theta power density was significantly higher in session 8 than in session 4 which is even more important for the HRV group than for the HRV/NFB group (see Figure 7). There is no significant interaction between conditions and groups, nor between sessions, conditions and groups.

### Significant Session Events and Participants’ Comments

Participants of the HRV/NFB reported a lack of perception of control of the EEG after the first training session. Participants described a lack of feedback with regards to the execution of the training and were skeptical about their capacity to control their brainwaves. At the end of the eight training sessions, 12 out of 15 participants reported that they glimpsed the process to their EEG activity and reached a state of flow. While most participants mastered the HRV biofeedback training before the fourth session, most of them expressed doubts about the potential generalizability of the NFB training in their daily life. Only two participants of the HRV/NFB group experienced difficulties performing the HRV training. While participants reported difficulties to maintain a constant paced breathing rate even after eight 5 min-sessions, most mentioned that the HRV training could be beneficial in their daily practice. Four of them reported trying to use the breathing technique outside the training environment. However, participants of the HRV group also reported boredom and that eight sessions were not necessary.

With regards to sessions’ observations by the experimenter, all participants of the HRV/NFB group were conscientiously trying to control their EEG activity as required by the training protocol. Nevertheless, some were sometimes using inappropriate strategies. One participant found that his SMR activity could increase by moving his forehead muscles. The experimenter had to reframe the participant.

## Discussion

In this randomized mixed design experiment, we aimed to investigate whether participants can intentionally modify the power densities of specifically selected EEG frequencies with eight NFB training sessions. We compared an HRV BFB training group and a combined HRV/NFB training group to assess the effect of the EEG training and the EEG frequency specificity. We also investigated these effects within and across training sessions, to map the potential dynamics of training evolution.

Our results showed that the EEG activity of all the participants was modified across and within sessions. However, quite surprisingly, the effect was not only limited to the trained frequency bands or the training modality. Within the HRV/NFB training group, untrained EEG frequencies have been significantly modified, unlike some of the trained frequencies. The original idea behind the neurofeedback training effect is that the changes should only be circumscribed to the trained EEG frequencies (Zoefel et al., 2011). The EEG frequencies which are not used as feedback frequencies should be independent and not affected by the NFB training. However, our results contrasted with this widely held hypothesis. Moreover, EEG activity was modified in both the HRV group and the HRV/NFB groups. Both theta and alpha amplitudes were both significantly higher in session 8 than in session 4. Moreover, these amplitude differences are more important for the HRV group than in the HRV/NFB group. These findings show that HRV BFB training can fulfill a mediating role for the NFB training as previously reported by Prinsloo et al. (2013). These authors found an impact of BFB training on theta (i.e., increase), beta (i.e., decrease) and the theta/beta ratio (i.e., increase) (Prinsloo et al., 2013). Furthermore, the effect of the HRV biofeedback training on the EEG activity favors the neurovisceral integration model (e.g., Friedman, 2007) identifying a flexible neural network associated with self-regulation and adaptability that might help the organism to respond effectively to demands from the environment (Friedman, 2007). Conversely, our results are not in favor of an effective operant conditioning paradigm which allows participants to wilfully target specific EEG activity during the training. By reporting learning indices (Ros et al., 2019), we showed that the non-specific components of the NFB training are more important than the training modality.

If the EEG changes are unrelated to the applied training, one potential mechanism subtending these changes is a placebo/Hawthorne effect. The use of a placebo or sham condition can rule out the Hawthorne effect (Dessy et al., 2018) and, as outlined by the best practices in the NFB experimental design, evaluate the efficacy of NFB training on the trained subjects (Ros et al., 2019). However, implementing a sham condition was not feasible in this study. Yet, the comparison between our two experimental groups (i.e., HRV biofeedback group and HRV/NFB group) offers us an alternative to the sham condition and helps us to investigate the specificity of the NFB training. A few previous SMR-based NFB pieces of research claimed to demonstrate the specificity of NFB by reporting only changes in the trained EEG frequencies and not the whole EEG spectrum (Egner and Gruzelier, 2004; Egner et al., 2004; Doppelmayr and Weber, 2011; Gruzelier, 2014; Schabus et al., 2014; Kober et al., 2015, 2017; Reichert et al., 2015). However, it remains unclear whether participants can intentionally modify amplitudes of specifically selected electroencephalographic frequencies. There is a difference between well-established relationships, such as muscle tension and chronic pain or, as we developed in our introduction, heart rate variability and anxiety, and our insights linking brain oscillations with psychological functioning (Thibault and Raz, 2016a, b, 2017). This difference does not allow us generalization and relates to a decades-old issue in psychophysiological research, being the lack of caution in causal interpretation (Cacioppo et al., 2007). Indeed, even if a person exhibiting high levels of SMR is in an optimal state of cognitive functioning, the SMR may not play a causal role in this state (Beyerstein, 1990). As showed by Beyerstein (1990), people were able to produce high levels of alpha waves even when under threat of mild electric shock which is quite far away from an activity leading to a relaxed state; which was elegantly summarized by the author with the following analogy: “opening an umbrella is not enough to make it rain.” Brain imaging can inform us when and where changes occur in the brain, it cannot explain how the brain generates our cognitive capacity. It can reveal the correlates of cognition, but not its causal mechanism.

Furthermore, in a typical NFB training set-up, individuals who are trained to self-regulate their EEG activity are feeding back in real-time about their EEG activity. The received feedback includes their EEG activity as well the electromyographical (EMG) artifacts produced by their body. The occurrence of facial EMG can impact beta and ocular EMG activity (e.g., eye blinks and eye movement) can appear as pseudo-theta. As revealed in our sessions’ observations, one participant used facial EMG interference to increase his SMR activity. As mentioned by La Marca et al. (2018), controlling for artifact has a large effect on measurable outcomes and can be beneficial to improve the robustness of the training (La Marca et al., 2018). Offline EEG data processing allowed us to filter out the significant EMG artifacts from the EEG signals, which is the results we present. However, during their NFB training, participants received feedback which is not only the reflection of their EEG activity, since it relies on raw signals. The potential benefits obtained after the NFB training cannot be attributed to training-specific modifications. This reinforces the importance of the non-specific components of the NFB training to explain the potential benefits of the NFB training. However, the potential effect of the training modalities may have been masked by the methodological and theoretical issues associated with neurofeedback. Moreover, although our sample was homogeneous, its size was still modest. Thus, we cannot exclude that future research might develop a reliable protocol elicitating changes in the targeted EEG frequency bands.

## Conclusion

The EEG activity of all our participants were modified across and within sessions. However, contrary to what the most recent neurofeedback literature suggests, these EEG changes were not only circumscribed to the trained frequency bands or to the training modality. As emphasized by the consortium of researchers (Ros et al., 2019), EEG data of NFB training should always be monitored, analyzed and reported session by session in both clinical and research settings, to highlight whether there is a specific effect of the training modality.

## Data Availability Statement

The datasets for this article are not publicly available because it is not in accordance with the ethical procedures of ICH-GCP and GDPR. Requests to access the datasets should be directed to the corresponding author.

## Ethics Statement

This study was carried out in accordance with the recommendations of the ICH-GCP. The protocol was approved by the Medical Ethics Committee of the UZ Brussel/VUB. All subjects gave written informed consent in accordance with the Declaration of Helsinki.

## Author Contributions

All the authors contributed to this manuscript. ED was the first author and the principal investigator of this study. MP contributed in the data analysis and helped in the writing process of this manuscript. OM advised the first author for the statistical analysis. AC was involved in the development of the neurofeedback and biofeedback training protocol. XN supervised the signal processing. NP supervised the study and contributed to all the writing process.

## Conflict of Interest

The authors declare that the research was conducted in the absence of any commercial or financial relationships that could be construed as a potential conflict of interest.

## References

[B1] Baevsky R. M. Baranov V. M. Chernikova A. G. Funtova I. I Pashenko A. V. Tank J. (2005). “Results of cardiorespiratory system autonomic regulation investigations during long term international space station missions?: experiment “pulse”,” in *Proceedings of 9th European Symposium on Life Sciences in Space, 26th Annual International Gravitational Physiology Meeting, Held 26 June – 1 July 2005*, Cologne, 2–3.

[B2] Beyerstein B. L. (1990). Brainscams: neuromythologies of the new age. *Int. J. Ment. Health* 19 27–36.

[B3] Cacioppo J. T. Tassinary L. G. Berntson G. G. (2007). “Psychophysiological science: interdisciplinary approaches to classic questions about the mind,” in *Handbook of Psychophysiology*, eds Cacioppo J. T. Tassinary L. G. Berntson G. G., (Cambridge: Cambridge University Press), 1–16. 10.1017/cbo9780511546396.001

[B4] Dessy E. Van Puyvelde M. Mairesse O. Neyt X. Pattyn N. (2018). Cognitive performance enhancement: do biofeedback and neurofeedback work? *J. Cogn. Enhanc.* 2 12–42. 10.1007/s41465-017-0039-y 17978869

[B5] Doppelmayr M. Weber E. (2011). Effects of SMR and theta/beta neurofeedback on reaction times, spatial abilities, and creativity. *J. Neurother.* 15 115–129. 10.1080/10874208.2011.570689 25566034

[B6] Egner T. Gruzelier J. H. (2004). EEG biofeedback of low beta band components: frequency-specific effects on variables of attention and event-related brain potentials. *Clin. Neurophysiol.* 115 131–139. 10.1016/s1388-2457(03)00353-5 14706480

[B7] Egner T. Zech T. F. Gruzelier J. H. (2004). The effects of neurofeedback training on the spectral topography of the electroencephalogram. *Clin. Neurophysiol.* 115 2452–2460. 10.1016/j.clinph.2004.05.033 15465432

[B8] Friedman B. (2007). An autonomic flexibility–neurovisceral integration model of anxiety and cardiac vagal tone. *Biol. Psychol.* 74 185–199. 10.1016/j.biopsycho.2005.08.009 17069959

[B9] Gruzelier J. H. (2014). EEG-neurofeedback for optimising performance. III: a review of methodological and theoretical considerations. *Neurosci. Biobehav. Rev.* 44 159–182. 10.1016/j.neubiorev.2014.03.015 24690579

[B10] Guyenet P. G. (2014). Regulation of breathing and autonomic outflows by chemoreceptors. *Compr. Physiol.* 4 1511–1562. 10.1002/cphy.c140004 25428853 PMC4794276

[B11] Hammond D. C. Bodenhamer-Davis G. Gluck G. Stokes D. Harper S. H. Trudeau D. (2011). Standards of practice for neurofeedback and neurotherapy: a position paper of the international society for neurofeedback & research. *J. Neurother.* 15 54–64. 10.1080/10874208.2010.545760

[B12] Kober S. E. Witte M. Neuper C. Wood G. (2017). Specific or nonspecific? Evaluation of band, baseline, and cognitive specificity of sensorimotor rhythm- and gamma-based neurofeedback. *Int. J. Psychophysiol.* 120 1–13. 10.1016/j.ijpsycho.2017.06.005 28652143

[B13] Kober S. E. Witte M. Stangl M. Väljamäe A. Neuper C. Wood G. (2015). Shutting_ down sensorimotor interference unblocks the networks for stimulus processing: an SMR neurofeedback training study. *Clin. Neurophysiol.* 126 82–95. 10.1016/j.clinph.2014.03.031 24794517

[B14] La Marca J. P. Cruz D. Fandino J. Cacciaguerra F. R. Fresco J. J. Guerra A. T. (2018). Evaluation of artifact-corrected electroencephalographic (EEG) training: a pilot study. *J. Neural. Transm.* 125 1087–1097. 10.1007/s00702-018-1877-1 29582150

[B15] Lehrer P. M. Gevirtz R. (2014). Heart rate variability biofeedback: how and why does it work? *Front. Psychol.* 5:756. 10.3389/fpsyg.2014.00756 25101026 PMC4104929

[B16] Lehrer P. M. Vaschillo E. Vaschillo B. (2000). Resonant frequency biofeedback training to increase cardiac variability?: rationale and manual for training. *Appl. Psychophysiol. Biofeedback* 25 177–191. 10999236 10.1023/a:1009554825745

[B17] Niv S. (2013). Clinical efficacy and potential mechanisms of neurofeedback. *Pers. Individ. Dif.* 54 676–686. 10.1016/j.paid.2012.11.037

[B18] Pattyn N. Mairesse O. Cortoos A. Morais J. Soetens E. Roelands B. (2014). Cardiac reactivity and preserved performance under stress: two sides of the same coin? *Int. J. Psychophysiol.* 93 30–37. 10.1016/j.ijpsycho.2013.03.008 23528304

[B19] Pattyn N. Migeotte P.-F. Neyt X. den Nest A. Van, Cluydts R. (2010). Comparing real-life and laboratory-induced stress reactivity on cardio-respiratory parameters: differentiation of a tonic and a phasic component. *Physiol. Behav.* 101 218–223. 10.1016/j.physbeh.2010.04.037 20451535

[B20] Pattyn N. Neyt X. Henderickx D. Soetens E. (2008). Psychophysiological investigation of vigilance decrement: boredom or cognitive fatigue? *Physiol. Behav.* 93 369–378. 10.1016/j.physbeh.2007.09.016 17999934

[B21] Prinsloo G. E. Rauch H. G. L. Karpul D. Derman W. E. (2013). The effect of a single session of short duration heart rate variability biofeedback on EEG: a pilot study. *Appl. Psychophysiol. Biofeedback* 38 45–56. 10.1007/s10484-012-9207-0 23129056

[B22] Reichert J. L. Kober S. E. Neuper C. Wood G. (2015). Resting-state sensorimotor rhythm (SMR) power predicts the ability to up-regulate SMR in an EEG-instrumental conditioning paradigm. *Clin. Neurophysiol.* 126 2068–2077. 10.1016/j.clinph.2014.09.032 25743268

[B23] Ros T. Enriquez-geppert S. Zotev V. Young K. Wood G. Wan F. (2019). Consensus on the reporting and experimental design of clinical and cognitive-behavioural neurofeedback studies (CRED-nf checklist). *PsyArXiv* [Preprint] Available at: https://psyarxiv.com/nyx84 (accessed January, 2019). 10.1093/brain/awaa009 PMC7296848 32176800

[B24] Ros T. Moseley M. J. Bloom P. A. Benjamin L. Parkinson L. A. Gruzelier J. H. (2009). Optimizing microsurgical skills with EEG neurofeedback. *BMC Neurosci.* 10:87. 10.1186/1471-2202-10-87 19630948 PMC2723116

[B25] Sanders A. F. (1983). Towards model of stress and performance. *Acta Psychol.* 53 61–97. 10.1016/0001-6918(83)90016-1 6869047

[B26] Schabus M. Griessenberger H. Gnjezda M.-T. Heib D. P. J. Wislowska M. Hoedlmoser K. (2017). Better than sham? A double-blind placebo-controlled neurofeedback study in primary insomnia. *Brain* 140 1041–1052. 10.1093/brain/awx011 28335000 PMC5382955

[B27] Schabus M. Heib D. P. J. Lechinger J. Griessenberger H. Klimesch W. Pawlizki A. (2014). Enhancing sleep quality and memory in insomnia using instrumental sensorimotor rhythm conditioning. *Biol. Psychol.* 95 126–134. 10.1016/j.biopsycho.2013.02.020 23548378

[B28] Schönenberg M. Wiedemann E. Schneidt A. Scheeff J. Logemann A. Keune P. M. (2017). Neurofeedback, sham neurofeedback, and cognitive-behavioural group therapy in adults with attention-deficit hyperactivity disorder: a triple-blind, randomised, controlled trial. *Lancet Psychiatry* 4 673–684. 10.1016/S2215-0366(17)30291-2 28803030

[B29] Thayer J. F. Hansen A. L. Saus-rose E. Johnsen B. H. (2009). Heart rate variability, prefrontal neural function, and cognitive performance?: the neurovisceral integration perspective on self-regulation, adaptation, and health. *Ann. Behav. Med.* 37 141–153. 10.1007/s12160-009-9101-z 19424767

[B30] Thayer J. F. Lane R. D. (2009). Claude Bernard and the heart–brain connection: further elaboration of a model of neurovisceral integration. *Neurosci. Biobehav. Rev.* 33 81–88. 10.1016/j.neubiorev.2008.08.004 18771686

[B31] Thibault R. T. Lifshitz M. Raz A. (2017). Neurofeedback or neuroplacebo? *Brain* 140 862–864. 10.1093/brain/awx033 28375458

[B32] Thibault R. T. MacPherson A. Lifshitz M. Roth R. R. Raz A. (2018). Neurofeedback with fMRI: a critical systematic review. *Neuroimage* 172 786–807. 10.1016/j.neuroimage.2017.12.071 29288868

[B33] Thibault R. T. Raz A. (2016a). Neurofeedback: the power of psychosocial therapeutics. *Lancet Psychiatry* 3 e18 10.1016/s2215-0366(16)30326-1 27794377

[B34] Thibault R. T. Raz A. (2016b). When can neurofeedback join the clinical armamentarium? *Lancet Psychiatry* 3 497–498. 10.1016/s2215-0366(16)30040-2 27262039

[B35] Thibault R. T. Raz A. (2017). The psychology of neurofeedback: clinical intervention even if applied placebo. *Am. Psychol.* 72 679–688. 10.1037/amp0000118 29016171

[B36] Thibault R. T. Raz A. (2018). A consensus framework for neurofeedback research (and the perils of unfounded neuroreductionism): reply to Micoulaud-Franchi and Fovet (2018). *Am. Psychol.* 73 936–937. 10.1037/amp0000366 30284894

[B37] Wilmore J. H. Costill D. L. (2004). *Physiology of Sport and Exercise*, 3rd Edn Champaign, IL: Human Kinetics.

[B38] Yucha C. Montgomery D. (2008). *Evidence-Based Practice in Biofeedback and Neurofeedback.* Wheat Ridge, CO: AAPB.

[B39] Zoefel B. Huster R. J. Herrmann C. S. (2011). Neurofeedback training of the upper alpha frequency band in EEG improves cognitive performance. *Neuroimage* 54 1427–1431. 10.1016/j.neuroimage.2010.08.078 20850552

